# LoLoPicker: detecting low allelic-fraction variants from low-quality cancer samples

**DOI:** 10.18632/oncotarget.16144

**Published:** 2017-03-12

**Authors:** Jian Carrot-Zhang, Jacek Majewski

**Affiliations:** ^1^ Cancer Program, The Broad Institute of Harvard and MIT, Cambridge, Massachusetts, USA; ^2^ Department of Medical Oncology, Dana-Farber Cancer Institute, Boston, Massachusetts, USA; ^3^ Department of Human Genetics, McGill University, Montreal, Quebec, Canada; ^4^ Genome Quebec Innovation Centre, Montreal, Quebec, Canada

**Keywords:** somatic mutation detection, low allelic-fraction variants, high specificity, FFPE samples

## Abstract

**Introduction:**

Although several programs are designed to identify variants with low allelic-fraction, further improvement is needed, especially to push the detection limit of low allelic-faction variants in low-quality, ”noisy” tumor samples.

**Results:**

We developed LoLoPicker, an efficient tool dedicated to calling somatic variants from next-generation sequencing (NGS) data of tumor sample against the matched normal sample plus a user-defined control panel of additional normal samples. The control panel allows accurately estimating background error rate and therefore ensures high-accuracy mutation detection.

**Conclusions:**

Compared to other methods, we showed a superior performance of LoLoPicker with significantly improved specificity. The algorithm of LoLoPicker is particularly useful for calling low allelic-fraction variants from low-quality cancer samples such as formalin-fixed and paraffin-embedded (FFPE) samples.

Implementation and Availability: The main scripts are implemented in Python-2.7 and the package is released athttps://github.com/jcarrotzhang/LoLoPicker.

## INTRODUCTION

The detection of somatic mutations in tumors remains challenging. One of the major problems is that variants with low allelic-fractions that are commonly observed in tumor samples owing to normal tissue contaminations or cancer heterogeneity, are difficult to distinguish from systematic errors inherent to most sequencing technologies. Moreover, technical artifacts, such as C to T and G to A transitions can arise from the formalin-fixation process, which is widely used to preserve tissues in hospitals worldwide [1, 2]. Therefore, additional filters against FFPE-specific errors are required [3].

NGS has emerged as an invaluable tool for discovering disease-causing genes. For many basic research or clinical laboratories, the number of samples being sequenced has increased dramatically. Some laboratories build their in-house databases to enable them filtering out false-positive calls that are specific to library preparation, protocols, instruments, environmental factors or analytical pipeline. Moreover, a panel of control samples provides an opportunity to precisely estimate background error rates, which can be used to increase the sensitivity of calling single nucleotide variants (SNVs) for sites with low error rate, and to reduce false positives for sites with high error rate [4].

**Figure 1:**
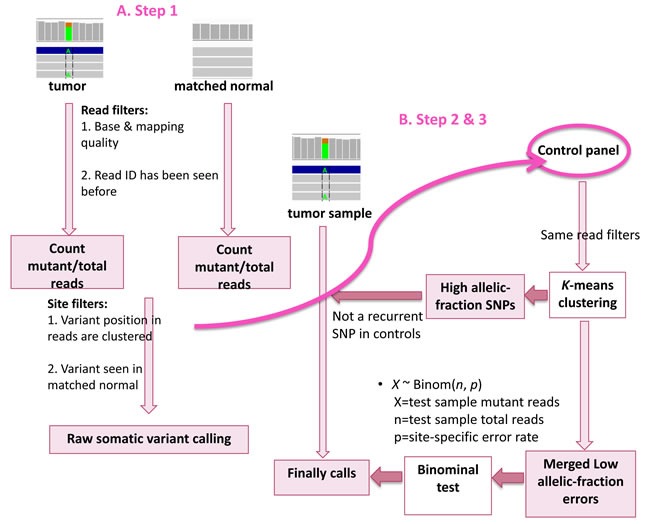
Workflow of LoLoPicker Step 1: LoLoPicker performs its raw somatic variant calling using tumor and its matched normal sample. Read filters and site filters are shown in order and listed in Table 1. Step 2: LoLoPicker takes the positions of the raw somatic variants identified from Step 1 and counts reads for each sites (same read filters applied) in each control sample provided in the user-defined control panel. Step 3: Where necessary, a K-means clustering is performed based on the allelic-fraction of the variant in each control sample, in order to identify control samples containing either germline variants or background errors at the given position. LoLoPicker then filters out recurrent germline variants identified from the control panel, merges the background errors and calculates the site-specific error rate. Finally, a binomial test followed by multiple-testing correction is performed.

Although several programs have been designed particularly to call somatic variants at low-allelic fraction, a caller that is able to detect low-allelic fraction variants in low-quality samples, such as FFPE samples is needed. Here we present LoLoPicker, a flexible variant caller for whole-exome sequencing (WES), whole-genome sequencing (WGS) and targeted re-sequencing analysis that can handle low-quality samples. This program allows users to provide a control panel consisting of normal samples processed using similar procedures as the tumor (e.g. FFPE samples). The control panel is used to calculate background error rate. Then, a binominal test is performed to determinate whether the tumor variant exceeds the background noise (Figure 1). LoLoPicker's algorithm allows genome-wide variant calling with hundreds of control samples in a few hours. A detailed description of the algorithm is described in the Materials and Methods section.

## RESULTS

### Benchmarking analysis

To access the performance of LoLoPicker in comparison to other variant callers, we benchmarked LoLoPicker, MuTect [5], VarScan [6] and LoFreq [7] against two datasets, a true positive dataset and a false positive dataset. For the dataset containing true positives, somatic SNVs identified from WES of an ovarian tumor and validated by Sanger sequencing were used (Supplementary Table 1). The WES analysis of the ovarian tumor and its matched germline sample was published in our previous work [8]. To access the performance of detecting low allelic-fraction variants, BAM files of the ovarian tumor and the germline sample were mixed to ensure that the true positives described above were present at low allelic-fraction. In the mixed BAM file, the total coverage of the variants ranged from 39 to 715; the alternative read-count of variants ranged from 3 to 61; the allelic-fraction of variants ranged from 1% to 14%. We ran the four mutation callers using the mixed sample as the “new” tumor sample, and the original blood sample as the matched normal sample. For specificity, a sample that underwent WES twice in two different batches was used. The BAM file produced from the first batch was used as the tumor sample, and the one from the second batch was used as the matched normal sample. Therefore, any variants called by the four programs were considered as false positives. An example of data used for the benchmarking analysis is available in the LoLoPicker Github repository and the scripts used to generate the results are included in the Supplementary Information.

**Figure 2:**
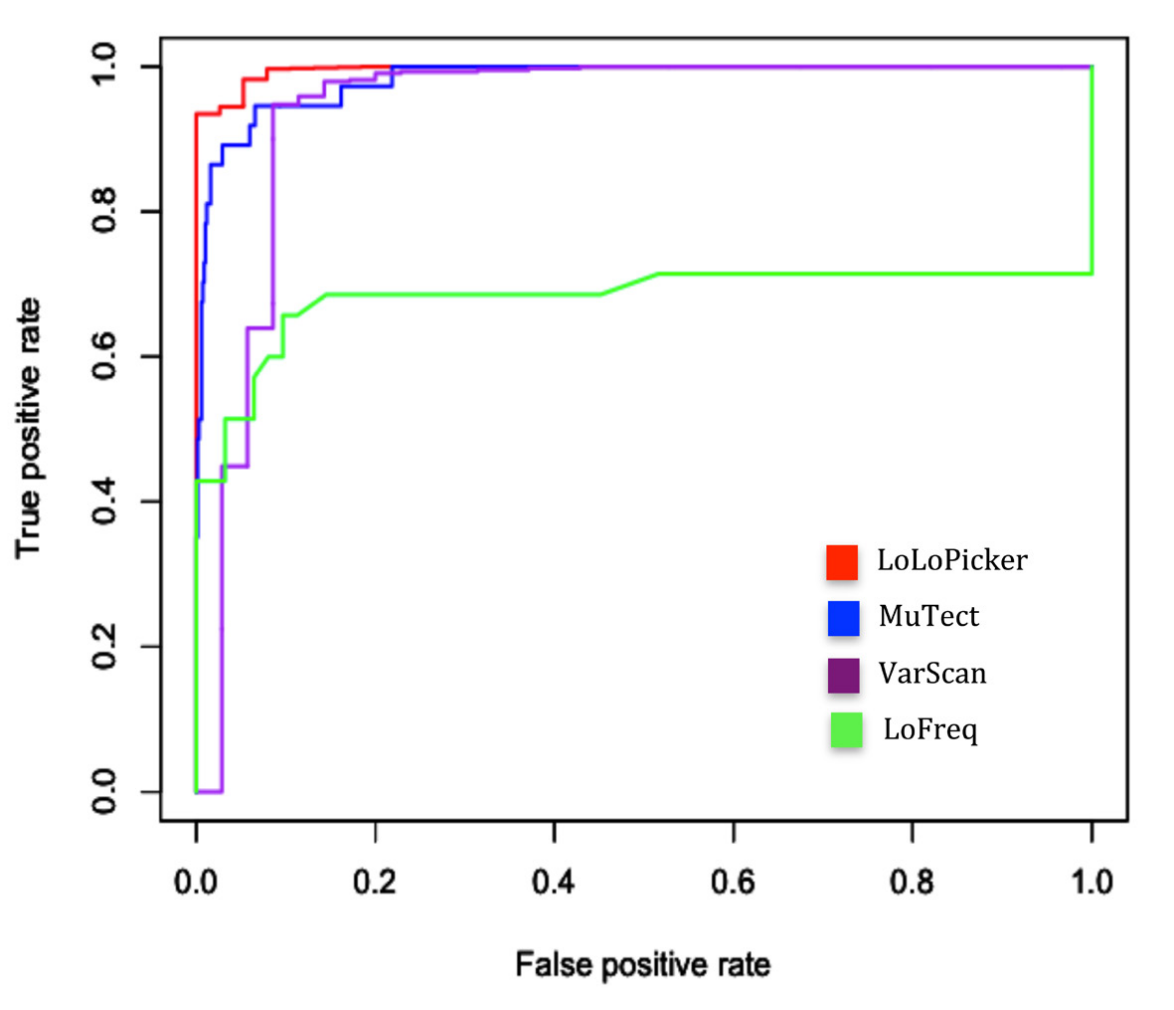
ROC analysis The performances of LoLoPicker, MuTect, VarScan, and LoFreq in calling SNVs at low allelic-fraction (1-10%) are compared using the benchmarked samples. LoLoPicker showed increased sensitivity and specificity compared to other methods. The ROC analysis is based on the binomial p-value of LoLoPicker, the tumor Fstar LOD score of MuTect, the somatic p-value of VarScan, and the VCF quality score of LoFreq.

Because samples used in this benchmarking analysis were fresh-frozen samples, we used 500 germline, non-cancer samples from unrelated individuals that were processed for WES in the same way as the fresh-frozen samples (as described in Materials and Methods) for our control panel. The control panel was also provided to MuTect to enable its panel of normal filtering mode. As suggested by MuTect, the “--normal_panel” option allows an additional filter from a panel of normal samples that can further improve MuTect's specificity [5]. For variant calling, default parameters were applied to all programs, except in VarScan, “--min-coverage 0”, and “--min-var-freq 0” were used to allow calling low allelic-fraction variants, and in MuTect, the “--normal_panel” option under MuTect's artifact detection mode was applied. The performances of the four mutation callers were visualized by ROC analysis based on the binomial p-value of LoLoPicker, the tumor Fstar LOD score of MuTect, the somatic p-value of VarScan, and the VCF quality score of LoFreq. The ROC analysis was conducted using the ROCR package [9]. As the results, both LoLoPicker and MuTect showed the highest sensitivity, and LoLoPicker showed the highest specificity compared to other programs (Figure 2, Table 1).

**Table 1:** Number of true positives and false positives called by LoLoPicker, MuTect, VarScan and LoFreq in the benchmarked analysis, suggesting high sensitivity and specificity of LoLoPicker

Tools	True Positives	False Positives
**LoLoPicker**	21/21	3
**MuTect**	21/21	25*
**VarScan**	18/21	21
**LoFreq**	18/21	53

*Number of false positives called by MuTect is reduced to 15, when we apply the “--normal_panel” option in MuTect using the same controls as LoLoPicker.

### Analyzing fresh-frozen tumor sample

We then applied LoLoPicker, MuTect and VarScan to analyze a real tumor sample with its matched germline sample from a glioblastoma (GBM) patient (GBM_9). Because the tumor sample is a fresh-frozen sample processed similarly to the benchmarked samples, we ran LoLoPicker and MuTect against the same control panel as used in the benchmarking analysis. The average coverage of GBM_9 was 59X for the tumor sample, and 78X for the matched blood sample. When parallelized in 8 threads, LoLoPicker finished analyzing the WES data of GBM_9 with its blood sample against 500 control samples in approximately 2.5 hours.

**Figure 3:**
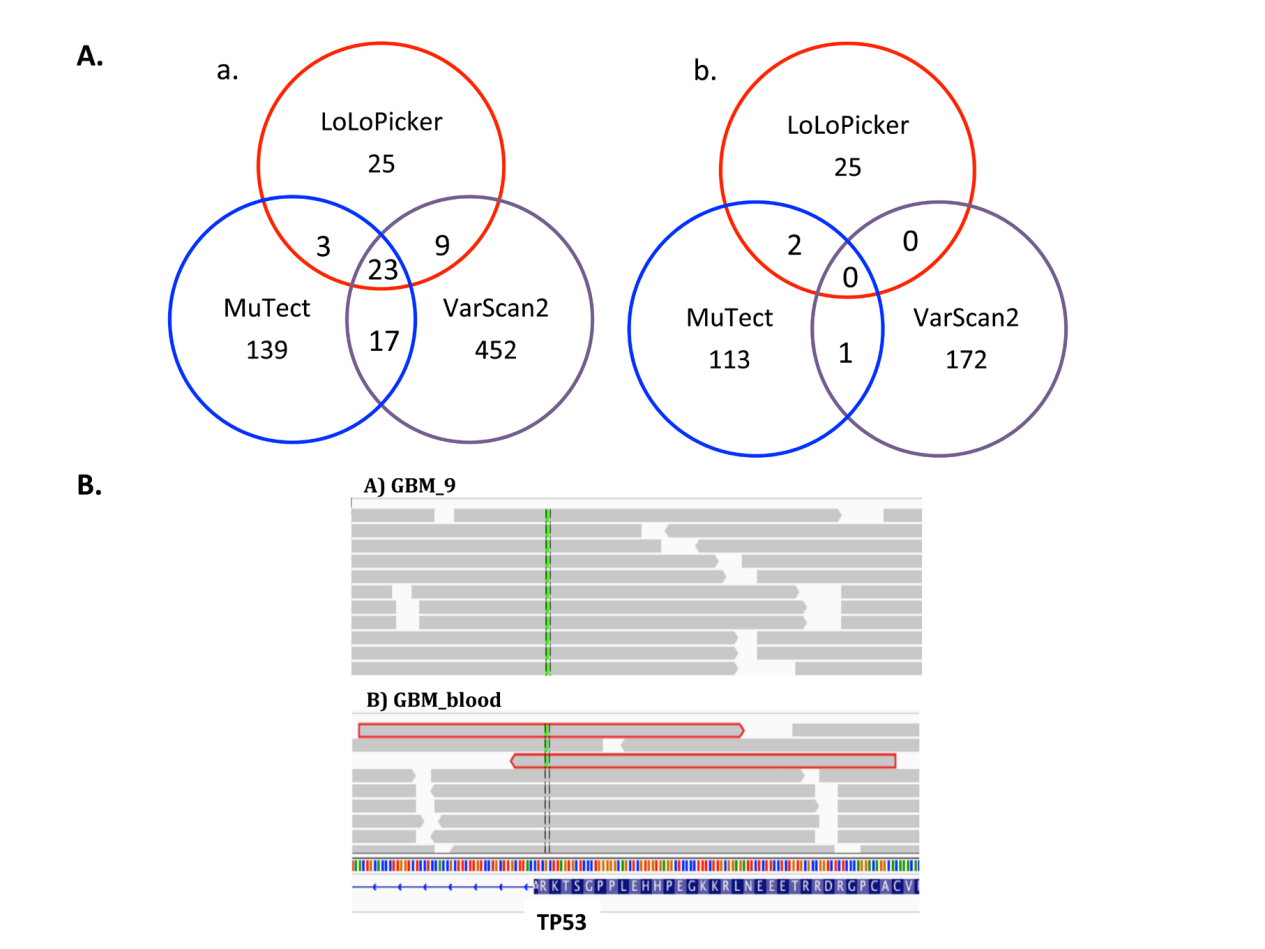
**A**. Venn diagram of variants in GBM_9 called from different tools. a. Number of total variants called by LoLoPicker, MuTect and VarScan. b. Number of low allelic-fraction (<10%) variants called by the three callers. **B**. Snapshot of IGV showing TP53 mutation (p.R174X) in GBM_9 and its matched blood sample. MuTect filtered out this mutation because three reads supporting the variant are observed in the blood sample. However, two of them were overlapping read-pair (highlighted in red), which are counted as one in LoLoPicker. The third read has low mapping quality less than 30. Therefore, this mutation is retained in LoLoPicker.

As the results, LoLoPicker called 60 somatic variants, while MuTect and VarScan called 182 variants and 503 variants, respectively. Most of the low allelic-fraction variants were not called by more than one method (Figure 3A). Known GBM driving mutations were identified, including p.R174X in TP53, p.K27M in H3F3A, p.R1480X in ATRX, and p.H1047R in PIK3CA. Only LoLoPicker successfully identified all four mutations. MuTect discarded the TP53 mutation because it found more than one read supporting the variant in the blood sample. The TP53 mutation is later validated as a real somatic mutation using targeted re-sequencing. LoLoPicker retained this mutation because we consider overlapping read-pair covering the same variant as one (Figure 3B). Notably, the PIK3CA mutation showed a low allelic-fraction at 6%. VarScan missed the PIK3CA mutation, suggesting again that the sensitivity of VarScan for detecting low-allelic fraction variants is not as high as LoLoPicker and MuTect.

We selected 14 SNVs identified from GBM_9. Variants that were called by both LoLoPicker and MuTect, or called by MuTect only but with potential implication in cancer were selected for targeted re-sequencing validation (Table 2). The goal of this validation is to assess whether LoLoPicker missed true variants. All the selected variants were not previously reported in public sequencing databases including dbSNP, 1000 genome and Exome Variant Server (EVS). We found that variants called by both programs were all validated; whereas variants called by MuTect but rejected by LoLoPicker were all failed validation, including four variants with higher coverage (>=5X) supporting the alternative base. Our results suggested the high specificity of LoLoPicker without rejecting true variants as a trade-off.

**Table 2:** Variants in GBM_9 selected for targeted re-sequencing validation and their status in LoLoPicker and MuTect calls

Position	Ref	Alt	Altered Reads	Total Reads	Allelic-fraction	Protein Change	Gene	LoLoPicker *p*_value and judgement	MuTect Judgement	MiSeq Validation
chr1:89521863	C	A	16	92	0.17	p.A402S	*GBP1*	5.30e-31 / real	KEEP	Yes
chr13:24895566	T	C	5	47	0.11	p.I221T	*C1QTNF9*	9.66e-06 / real	KEEP	Yes
chr3:178952085	A	G	5	94	0.06	p.H1047R	*PIK3CA*	2.62e-05 / real	KEEP	Yes
chr9:5231708	G	A	4	22	0.17	p.G62E	*INSL4*	2.04e-04 / real	KEEP	Yes
chr11:71907000	C	A	4	65	0.06	p.P185T	*FOLR1*	0.32 / reject	KEEP	No
chr13:25378544	C	A	5	75	0.07	p.P690T	*RNF17*	0.6 / reject	KEEP	No
chr19:40580859	C	A	4	68	0.06	p.G497V	*ZNF780A*	1 / reject	KEEP	No
chrX:3240813	C	A	3	31	0.09	p.E971D	*MXRA5*	1 / reject	KEEP	No
chr1:65301884	C	A	3	44	0.06	p.C1052F	*JAK1*	1 / reject	KEEP	No
chr2:62099221	C	A	3	29	0.1	p.R466L	*CCT4*	1 / reject	KEEP	No
chr15:22742690	T	C	8	121	0.07	p.W359R	*GOLGA6L1*	1 / reject	KEEP	No
chr3:4715013	A	C	7	23	0.3	p.T785P	*ITPR1*	1 / reject	KEEP	No
chr18:77805926	T	G	5	13	0.38	p.W240G	*RBFA*	1 / reject	KEEP	No
chr6:116912080	C	A	3	58	0.05	p.L193I	*RWDD1*	1 / reject	KEEP	No

All selected variants are not previously reported in public databases.

**Figure 4:**
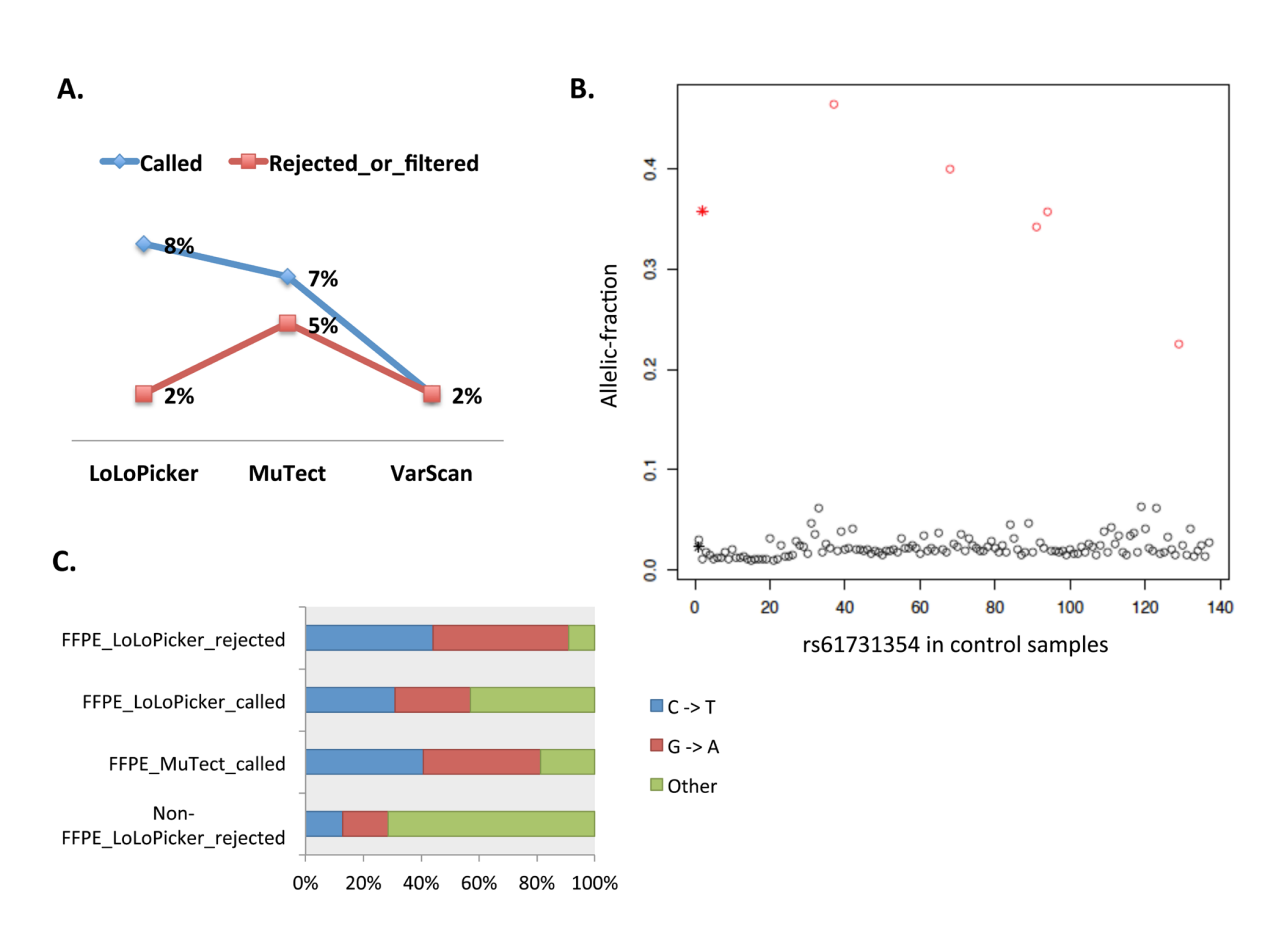
**A**. Proportion of variants reported in COSMIC. Variants used in this plot are either called or rejected by LoLoPicker, MuTect or VarScan in GBM_9. **B**. K-means clustering distinguishes germline variants from background noise. Identified germline variants (red circles) and background errors (black circles) from 500 germline, non-cancer samples. At this position, one SNP (rs61731354) is found in five samples with mean allelic-fraction at 0.35 (red star). Sequencing artifacts are found in 130 samples at the site with mean allelic-fraction at 0.02 (black star). **C**. Percentage of C to T and G to A transitions in LoLoPicker and MuTect produced results. 91% of the variants rejected by LoLoPicker in UN5 (FFPE_LoLoPicker_rejected) are C to T and G to A transitions; whereas 57% of the variants called by LoLoPicker (FFPE_LoLoPicker_called) are these transitions. By contrast, 81% of the variants called by MuTect in UN5 are C to T and G to A transitions (FFPE_MuTect_called). These transitions are known FFPE artifacts and are enriched in our FFPE sample compared to our fresh-frozen sample (non-FFPE_LoLoPicker_rejected), suggesting the necessity of using LoLoPicker's statistical framework to reduce false positives related to batch effects.

Other somatic variants may exist in GBM_9. Variants reported in the Catalogue of Somatic Mutations in Cancer (COSMIC) were enriched among variants called by LoLoPicker (10%) compared to 2% of LoLoPicker-rejected calls. By contrast, among the variants called by MuTect and VarScan, 7% and 2% were reported in COSMIC, respectively. MuTect also rejected 5% COSMIC reported variants (Figure 4A). This suggested that compared to MuTect and VarScan, LoLoPicker called more cancer-related variants. Moreover, LoLoPicker identified 131 germline variants in GBM_9 from the control panel of 500 non-cancer, germine samples (Figure 4B). All these variants were previously reported in the dbSNP database.

### Analyzing FFPE tissue

In our previously published work on small cell carcinoma of the ovary, hypercalcemic type (SCCOHT), we showed that only one gene – SMARCA4 – was recurrently mutated in our case cohort and was responsible for this disease [10]. Because the SCCOHT samples have a very simple spectrum of somatic mutations, they are ideal for identifying FFPE-related problems. Therefore, we tested LoLoPicker, MuTect and VarScan on the tumor and its matched normal tissue of an FFPE-SCCOHT sample (UN5) sequenced by WES. A control panel consisting 35 FFPE samples was provided to both LoLoPicker and MuTect. All the three programs called the SMARCA4 mutation (c.2275-1 G>T). Although very few variants other than the SMARCA4 mutation were expected, MuTect and VarScan called 483 and 143 variants, respectively. Moreover, MuTect only filtered out 19 variants using its ““--normal_panel” option. By contrast, LoLoPicker identified 87 variants. After filtering out dbSNP reported variants, 42 variants were retained in LoLoPicker, whereas 384 and 101 variants were retained in MuTect and VarScan, respectively. Among the 42 variants called by LoLoPicker, 21 (50%) of them were C to T or G to A transitions; whereas 82% of the variants called by MuTect were C to T or G to A. Moreover, most of the LoLoPicker rejected calls (91%) were these transitions (Figure 4C). These transitions are possible artifacts related to the FFPE protocol [2,3].

## DISCUSSION

LoLoPicker is tailored for calling low allelic-fraction, somatic variants. Compared to other programs, LoLoPicker showed the highest sensitivity and more importantly, a dramatically improved specificity, thus emphasizing the importance of precisely measuring the background error rate from additional control samples, rather than from the matched normal sample solely. Compared to MuTect's method that ruling out any variants or artifacts seen more than once in the control panel, LoLoPicker's statistical framework allowed higher specificity and filtered out more FFPE-related artifacts. Although we mainly demonstrated the performance of LoLoPicker in WES analysis, this program is also feasible for WGS and targeted re-sequencing analysis. Recommended parameters for analyzing WGS and targeted re-sequencing data are provided in the Supplementary Information. The runtime of LoLoPicker depends on the sample size of the control panel. Notably, the LoLoPicker algorithm can be easily parallelized to allow genome-wide variant calling against hundreds of control samples in a few hours.

Samples provided in the control panel are essential in estimating the background error rate. The more normal samples are included in the control panel, the more accurate error rate will be measured. However, we suggest that samples processed and aligned similarly to the tumor sample should be used. For example, using 500 control samples that processed similarly to the fresh-frozen tumor samples, we reduced the false calls to 3 in the benchmarked dataset, and we rejected all the variants that were not validated by targeted re-sequencing but were called by MuTect in GBM_9. However, using this control panel, LoLoPicker called more false positives in the FFPE sample, UN5 (112 false calls, considering the only cancer-driving event was the SMARCA4 mutation), compared to 86 false calls (before filtering out dbSNP variants) using FFPE samples as our controls, even though the sample size of the FFPE-control panel was as small as 35.

Although the specificity of LoLoPicker was significantly improved compared to other programs, there were false positive calls retained in LoLoPicker generated results. In the benchmarking analysis, the three false positives were all previously reported in the dbSNP database, suggesting that they might be germline variants from foreign DNA contamination. LoLoPicker could not filter out these variants, because they were not recurrently present in the control panel. Therefore, we suggest that as the number of control samples available for a single research group is usually limited, the final calls of LoLoPicker should be further filtered against public databases, such as dbSNP, 1000 genome, EVS and ExAC. Additional filter against public databases also identified more germline variants in GBM_9 and UN5 which were missed from the control panel. Moreover, the increased number of false positive calls in the FFPE sample analysis was associated with the lack of sufficient support from the control panel – even though LoLoPicker merged all the reads from 35 samples, some sites were not well covered in the control panel due to the exome-capture efficiency bias and therefore, LoLoPicker was not able to identify potential artifacts in some regions. The performance of LoLoPicker in the FFPE analysis might be further improved, if we were able to obtain more FFPE-normal samples for the control panel. Finally, we notice that our current model is not suitable for indel calling and therefore, further efforts should be made to develop novel methods, for instance, a machine learning algorithm [11] in order to detect low fraction, somatic indels.

In conclusion, we showed that our new somatic mutation caller, LoLoPicker had superior performance compared to other popular callers, particularly with significantly improved specificity in calling low-allelic fraction variants. This program can handle WES, WGS and targeted re-sequencing data, as long as the users have control samples that are sequenced and aligned in the same way as the test tumor sample. The variant calling results can be improved with a smaller sample size of the control panel (e.g. 35 controls used in the FFPE analysis). When the sample size is large (e.g. 500 germline samples used in the fresh-frozen sample analysis), LoLoPicker can finish the analysis in a few hours on a dual-core machine. LoLoPicker is particularly useful for calling variants from FFPE samples. As FFPE samples are commonly used, our method will significantly benefit the current clinical cancer research.

## MATERIALS AND METHODS

### Step 1: raw somatic variant calling

In order to identify raw, somatic variants, LoLoPicker first walks through the tumor and its matched normal sample sequences and identifies sites with reads containing non-reference bases using pysamstats (https://github.com/alimanfoo/pysamstats). Then for each of these potential variants, LoLoPicker performs two classes of filtering: read filtering and site filtering (Table 3). After filtering out low base/mapping quality reads, overlapping read-pair covering the same variant, meaning that they sequence a variant from the same DNA fragment, are counted as one. This allows us to remove poorly supported variants for WES or WGS analysis, where sequencing depth can be low in some regions. For example, variants supported by only two reads and both reads are from the same pair are filtered.

**Table 3:** LoLoPicker's filtering steps in order and the default settings applied in WES analysis

	Class	Default Thresholds
**Base quality**	Read filter	Base quality score <30
**Mapping quality**	Read filter	Mapping quality score < 30
**Overlapping read pairs**	Read filter	Read mate is previously counted
**Position in read**	Site filter	In either forward or reverse strand, all alternative bases are located within the first or last five read positions
**Variant in matched normal**	Site filter	Number of altered reads >= 2 in the matched normal sample

### Step 2: inspecting the control cohort

At a given site where a raw, somatic variant is observed in the tumor sample from Step 1, LoLoPicker takes the user-provided panel of control samples from unrelated, non-cancer individuals and counts reads for each control sample at this position along with the same read filters as described in Table 3. The allelic-fraction of the variant is subsequently calculated in each control sample.

### Step 3: core statistical framework

Again, at a given site where a variant is observed in the tumor sample, LoLoPicker chooses samples from the control panel that contain background errors (e.g. we select samples with an allelic-fraction of the variant typically less than 10%, because we consider that most low allelic-fraction variants in germline, non-cancer samples are unlikely to be real variants). If the variant is present at high allelic-fraction (>10%) in any control sample, a K-means clustering is performed on the site based on the variant allelic-fraction of all control samples in the panel. We consider control samples in the cluster with larger mean containing germline variants, and control samples in the cluster with smaller mean containing low-fraction artifacts. If the variant is observed recurrently in the control panel as a germline variant, this variant is unlikely to be important for cancer development and is therefore flagged and excluded from further consideration. The K-means clustering is performed on all sites independently.

LoLoPicker then merges the reads from all the background errors identified from the control panel. These errors are used to calculate a site-specific error rate. The error rate is calculated as the ratio of the total number of reads supporting the alternative base and the total number of reads covering the site:

$$p=\frac{\sum^{\text{​}}alternative\;read\;count\;of\;each\;control}{\sum^{\text{​}}total\;read\;count\;of\;each\;control}$$

We model the read count of a specific site to be distributed by a binomial distribution:

*X* ~ Binom(*n,* p)

*X* = number of reads supporting the alternative base in the tumor sample; n = number of total reads in the tumor sample; p = site-specific error rate calculated from the control panel.

A binomial test is performed, followed by multiple-testing correction (Bonferroni) with a cut-off of 0.05 for significance. To assess whether a site is sufficiently covered to be sensitive enough to detect a mutation, the statistical power of the binomial test of each site is calculated. We keep sites that are covered with at least 80% power to detect a mutation with 30% allelic-fraction for the tumor sample, and a mutation with 50% allelic-fraction for the matched normal sample, considering that the allelic-fraction of a heterozygous mutation in the tumor sample can be reduced owing to normal tissue contamination.

### Sequencing and alignments

Whole-exome library preparation, exome capture and sequencing were performed using our standard protocols at the McGill University and Génome Québec Innovation Centre. For germline and fresh-frozen tumor samples, the Agilent SureSelect kits were used, following the manufacturer's protocols. FFPE tissue-derived DNA was captured using the Nextera Rapid-Capture Exome kit. The paired-end sequencing was performed using Illumina HiSeq 2000 with 100-bp length. High-quality trimmed reads were aligned to the UCSC hg19 reference genome with Burrows-Wheeler aligner (BWA) version 0.5.9 [12]. Indels were re-aligned using the Genome Analysis Toolkit (GATK) IndelRealigner [13]. Reads marked as PCR duplicates by Picard were excluded from further analysis (http://picard.sourceforge.net/). To ensure the best performance of MuTect and LoFreq, GATK BaseRecalibrator was used to increase the base quality score accuracy. Targeted re-sequencing was performed using a MiSeq sequencing platform with an average coverage of 5000X. Primers used in the experiments were shown in Supplementary Table 2. Finally, a Fisher's exact test was performed to identify somatic variants from the tumor and the matched germline sample.

## SUPPLEMENTARY MATERIALS TABLES


