# Supplementary material for: LoLoPicker: detecting low allelic-fraction variants from low-quality cancer samples
Source: Oncotarget. 2017 Mar 12;8(23):37032–40. doi: 10.18632/oncotarget.16144 (PMC5514890; doi:10.18632/oncotarget.16144)
Supplement: Supplementary file 1 [file oncotarget-08-37032-s001.pdf]

LoLoPicker: detecting low allelic-fraction variants from low-quality cancer samples

Supplementary Materials

1. How to run the test sample and reproduce the results provided in the folder of OVCA_output in LoLoPicker_example.zip.

```
$ cd LoLoPicker

$unzip LoLoPicker_example.zip

$mkdir LoLoPicker_example/your_output


$python lolopicker/scripts/LoLoPicker_somatic.py \

-t LoLoPicker_example/example_OVCA.bam \

-n LoLoPicker_example/blood.bam \

-r LoLoPicker_example/region.fa \

-b LoLoPicker_example/example_interval.bed \

-o LoLoPicker_example/your_output


$python lolopicker/scripts/LoLoPicker_control.py \

-l LoLoPicker_example/samplelist.txt \

-r LoLoPicker_example/region.fa \

-n 4 \

-o LoLoPicker_example/your_output


$python lolopicker/scripts/LoLoPicker_stats.py
```

**-o LoLoPicker_example/OVCA_output **

--intervalsize 900*

*This option is used because provided bam file is truncated. Default setting is 30000000 for WES analysis.

After running LoLoPicker, four files can be found in the output folder. The final results are shown in file stats_calls.txt and filtered variants are shown in reject_calls.txt.

2. How to run targeted re-sequencing data.

$python lolopicker/scripts/LoLoPicker_somatic.py -t tumor.bam -n normal.bam -r reference.fa -b interval.BED -o outputpath

--normalalteredreads 50 --tumoralteredreads 50 --tumoralteredratio 0.005

$python lolopicker/scripts/LoLoPicker_control.py -l samplelist.txt -r reference.fa -n 8 -o output-path

$python lolopicker/scripts/LoLoPicker_stats.py -o outputpath --intervalsize length_of_target-ed_region

3. How to run WGS data.

Step one:

$python lolopicker/scripts/LoLoPicker_somatic.py -t tumor.bam -n normal.bam -r reference.fa -b interval_chr1.BED -o outputpathChr1

$python lolopicker/scripts/LoLoPicker_somatic.py -t tumor.bam -n normal.bam -r reference.fa -b interval_chr2.BED -o outputpathChr2

……

$python lolopicker/scripts/LoLoPicker_somatic.py -t tumor.bam -n normal.bam -r reference.fa -b interval_chrX.BED -o outputpathChrX

Step two:

$python lolopicker/scripts/LoLoPicker_control.py -l samplelist.txt -r reference.fa -n 8 -o output-pathChr1

$python lolopicker/scripts/LoLoPicker_control.py -l samplelist.txt -r reference.fa -n 8 -o output-pathChr2

……

$python lolopicker/scripts/LoLoPicker_control.py -l samplelist.txt -r reference.fa -n 8 -o output-pathChrX

$cat outputpathChr*/control_stats.txt >outputpathFinal/control_stats.txt

Step three:

$python lolopicker/scripts/LoLoPicker_stats.py -o outputpathFinal --intervalsize 3000000000

Supplementary Table S1: Status of true positives used for benchmarking analysis.

Position	Gene	Mutation	Reference coverage	Altered_base coverage	Allelic-fraction
chr10:106124579	*CCDC147*	p.A177S	185	25	0.12
chr17:38173081	*CSF3*	p.P162S	89	8	0.08
chr15:64496758	*CSNK1G1*	p.R294T	301	46	0.13
chr17:11696980	*DNAH9*	p.D2741A	277	31	0.10
chr4:88533803	*DSPP*	p.N155K	202	21	0.09
chr20:33874597	*FAM83C*	p.T662M	139	8	0.05
chr6:5369392	*FARS2*	p.V197M	53	4	0.07
chr14:25076412	*GZMH*	p.Y180X	428	6	0.01
chr10:126477647	*METTL10*	p.I86V	446	28	0.06262
chrX:153040228	*PLXNB3*	p.G1323R	39	3	0.07
chr12:3692299	*PRMT8*	p.D302N	124	19	0.13
chr2:65316194	*RAB1A*	p.N100S	715	52	0.07
chr7:122338859	*RNA133*	p.W38X	422	34	0.07
chrX:30870990	*TAB3*	p.E539K	262	11	0.04
chr1:234565362	*TARBP1*	p.D891N	385	61	0.14
chr17:7579358	*TP53*	p.R110P	161	10	0.06
chr7:158824649	*VIPR2*	p.L361M	116	6	0.05
chr16:72828578	*ZFHX3*	p.R1754Q	428	34	0.07
chr19:58420819	*ZNF417*	p.S276C	113	11	0.09
chr17:29554310	*NF1*	Splicing	172	12	0.07
chr19:46192605	*SNRPD2*	Splicing	128	21	0.14

An ovarian tumor with validated somatic mutations were merged with its matched blood sample.

Supplementary Table S2: Primers used for targeted re-sequencing validation

Position	Gene	Forward	Reverse
chr1:89521863	GBP1	ACACTGACGACATGGTTC-TACAGCTTGGTCACCTTG-GTGTTT	TACGGTAGCAGAGACTTG-GTCTCATTAAAGGCCCAGC-TAGAAAA
chr13:24895566	C1QTNF9	ACACTGACGACATGGTTC-TACACAGGGTGAGCCAG-GAGTC	TACGGTAGCAGAGACTTG-GTCTAGGCCTGGTCCT-CAGAGC
chr3:178952085	PIK3CA	ACACTGACGACATG-GTTCTACAATGATGCTTG-GCTCTGGAAT	TACGGTAGCAGAGACTTG-GTCTCAATTCCTATGCAATC-GGTCT
chr9:5231708	INSL4	ACACTGACGACATGGTTC-TACACCCATGCCTGAGA-AGACATT	TACGGTAGCAGAGACTTG-GTCTCCCATGAGATTTCTG-GTGAGA
chr11:71907000	FOLR1	ACACTGACGACATGGTTC-TACAGGCTGGCAGACCT-CAAGATA	TACGGTAGCAGAGACTTG-GTCTTCATGGCTGCAG-CATAGAAC
chr13:25378544	RNF17	ACACTGACGACATGGTTC-TACATCATCCACCTATTTT-GCCTAAAG	TACGGTAGCAGAGACTTG-GTCTAAATCATATAAACTT-GTTTGAAGTTGC
chr19:40580859	ZNF780A	ACACTGACGACATGGTTC-TACAGAGTTTTCTGAT-GTTGGGAAAG	TACGGTAGCAGAGACTTG-GTCTTCCAATGAGA-AACCTTTTGTATG
chrX:3240813	MXRA5	ACACTGACGACATG-GTTCTACAAAGGTGTG-CAAAGGTGTCTTC	TACGGTAGCAGAGA-CTTGGTCTTGAAC-CATCTCCTACTCTGCAC
chr1:65301884	JAK1	ACACTGACGACATGGTTC-TACACAGCCATGGGAC-TAGAATCTG	TACGGTAGCAGAGACTTG-GTCTACCATAGCAGCGTATA-CATGG
chr2:62099221	CCT4	ACACTGACGACATGGTTC-TACAATGCAGCAGGCCT-CATATTT	TACGGTAGCAGAGACTTG-GTCTTGCTTTTGCAGATGC-TATGG
chr15:22742690	GOL-GA6L1	ACACTGACGACATGGTTC-TACAGAACCAGCAACAG-GAGGAGA	TACGGTAGCAGAGACTTG-GTCTTGCATCTTCTCTTC-CAGCTCC
chr3:4715013	ITPR1	ACACTGACGACATG-GTTCTACAGATATCAGCT-GAACCTCTTTGC	TACGGTAGCAGAGACTTG-GTCTGGCTATCTACTGCCG-CACA
chr18:77805926	RBFA	ACACTGACGACATGGTTC-TACAGTGCTTGGTGT-GAAGCCTCT	TACGGTAGCAGAGA-CTTGGTCTTCTGCCTC-CAACTCCTCTGT
chr6:116912080	RWDD1	ACACTGACGACATG-GTTCTACAGCAGATA-CATTTCATATGCCACTT	TACGGTAGCAGAGACTTG-GTCTTGGAATTCTACTAT-TATCTTACCATCC
chr17:7577022	TP53	ACACTGACGACATG-GTTCTACACTTCTTTG-GCTGGGGAGAG	TACGGTAGCAGAGACTTG-GTCTGGGACAGGTAG-GACCTGATTT
